# Tumor suppressor XAF1 induces apoptosis, inhibits angiogenesis and inhibits tumor growth in hepatocellular carcinoma

**DOI:** 10.18632/oncotarget.2114

**Published:** 2014-06-18

**Authors:** Li Ming Zhu, Dong Mei Shi, Qiang Dai, Xiao Jiao Cheng, Wei Yan Yao, Ping Hu Sun, Yan Fei Ding, Min Min Qiao, Yun Lin Wu, Shi Hu Jiang, Shui Ping Tu

**Affiliations:** ^1^ Department of Gastroenterology, No.3 People's Hospital Affiliated to Shanghai Jiaotong University School of Medicine, Shanghai, China; ^2^ Department of Gastroenterology, Ruijin Hospital, Shanghai Jiaotong University School of Medicine, Shanghai, China; ^3^ Department of Gastroenterology, Huadong Hospital, Shanghai Fudan University, Shanghai, China

**Keywords:** XAF1, Hepatocellular cancer, Apoptosis, Angiogenesis, VEGF

## Abstract

X-linked inhibitor of apoptosis (XIAP)-associated factor 1 (XAF1), a XIAP-binding protein, is a tumor suppressor gene. XAF1 was silent or expressed lowly in most human malignant tumors. However, the role of XAF1 in hepatocellular carcinoma (HCC) remains unknown. In this study, we investigated the effect of XAF1 on tumor growth and angiogenesis in hepatocellular cancer cells. Our results showed that XAF1 expression was lower in HCC cell lines SMMC-7721, Hep G2 and BEL-7404 and liver cancer tissues than that in paired non-cancer liver tissues. Adenovirus-mediated XAF1 expression (Ad5/F35-XAF1) significantly inhibited cell proliferation and induced apoptosis in HCC cells in dose- and time- dependent manners. Infection of Ad5/F35-XAF1 induced cleavage of caspase -3, -8, -9 and PARP in HCC cells. Furthermore, Ad5/F35-XAF1 treatment significantly suppressed tumor growth in a xenograft model of liver cancer cells. Western Blot and immunohistochemistry staining showed that Ad5/F35-XAF1 treatment suppressed expression of vascular endothelial growth factor (VEGF), which is associated with tumor angiogenesis, in cancer cells and xenograft tumor tissues. Moreover, Ad5/F35-XAF1 treatment prolonged the survival of tumor-bearing mice. Our results demonstrate that XAF1 inhibits tumor growth by inducing apoptosis and inhibiting tumor angiogenesis. XAF1 may be a promising target for liver cancer treatment.

## INTRODUCTION

Hepatocellular carcinoma (HCC) is involved in multiple gene alterations including tumor suppressor inactivation, oncogene activation and apoptosis-related gene dysregulation [1]. Many studies have shown that inhibition of apoptosis plays an important role in tumor growth and drug resistance [2]. Inhibitor of Apoptosis (IAP) is identified as a family of endogenous inhibitors of caspases [3, 4]. IAPs are characterized by highly conserved Baculoviral IAP Repeats (BIR) that inhibit apoptosis and include 8 members [5]. X-linked IAP (XIAP) is the most potent member of human IAPs that inhibit the role of caspases [6]. XIAP directly binds to caspase-3, -7, -9 and prevents their activities to initiate or execute apoptotic pathways [7]. XIAP has been shown to be overexpressed in most human cancer cell lines and cancer tissues including HCC tissues. Its overexpression has been demonstrated to be the independent factor for predicting the poor prognosis of HCC patients after liver transplantation [8]. Studies have shown that XIAP antisense nucleic acid and small molecule inhibitors of XIAP induce apoptosis and inhibit tumor growth in HCC cells [9], indicating that targeting inhibition of XIAP may be a new approach for HCC therapy [10].

HCC has been recognized as a hypervascular cancer, but their vasculature type is not uniform. Small-sized and well-differentiated HCC generally show very few tumor vessels, whereas advanced HCC exhibit rich blood vessels [11]. Several factors participating in the development of microvasculature have been identified. Vascular endothelial growth factor (VEGF) is well established as one of the key regulators of angiogenesis [12]. VEGF activates VEGF-receptor (VEGFR), resulting in triggering network of VEGFR signaling pathways that promote endothelial cell growth, migration, and survival from pre-existing vasculature. VEGF also mediates vessel permeability that has been shown to be associated with malignant effusions and mobilizes endothelial progenitor cells from the bone marrow to distant sites of neovascularization [12]. Studies have shown that VEGF are strongly involved in the development of liver tumor neovascularization and the infiltration of cancer cells into the tumor capsule in HCC [13]. VEGF overexpression is correlated with the clinicopathological features of HCC [14]. The well-established role of VEGF in promoting tumor angiogenesis has led to the development of agents that selectively target VEGF pathway [15]. Therefore, the suppression of VEGF expression may provide a novel strategy in the treatment of HCC [11].

XIAP-associated factor 1 (XAF1) is identified as a XIAP-binding protein and can directly bind preferentially to XIAP BIR2 and antagonize the anti-caspase activity of XIAP to induce apoptosis [16]. XAF1 triggers the re-localization of XIAP from the cytosol to the nucleus, then sequester XIAP. Unlike the overexpression of XIAP in most human cancer tissues, XAF1 is ubiquitously expressed in normal and fetal tissues but weakly expressed or even undetectable in most human cancer cell lines [17] and human cancer tissues including gastric [18], colon [19] and pancreatic cancer [20]. Loss of XAF1 expression is correlated strongly with tumor staging and progression in human cancers [20, 21]. Loss of heterozygosity of XAF1 gene has been reported in human colorectal cancers [22] and promoter CpG hypermethylation of XAF1 has been found in several human malignant tumors such as gastric [18, 23], colon [23], melanoma [24] and urogenital tumor [25-27]. A recent report showed that a significantly low XAF1 expression in poorly differentiated HCC was related to the resistance to apoptosis [28].

Our previous studies have shown that XAF1 induced apoptosis through intrinsic and extrinsic apoptosis pathways in gastric and colon cancer cells [29, 30]. We have found that XAF1 induces autophagy by upregulating beclin 1 and inhibiting AKT pathway [31]. Recent studies have shown that p53 can suppress the transcription of XAF1 by interacting with a high affinity responsive element within XAF1 promoter in gastrointestinal cancer cells [32]. Studies have shown that XAF1 inhibits the migration of endothelial cells [33] and degrades survivin [34]. Previous studies have shown that overexpression of survivin is associated with angiogenesis [35, 36]. Our and other have demonstrated that inhibition of suvivin suppress tumor angiogenesis [37, 38]. However, whether XAF1 inhibits tumor angiogenesis remains unknown. In this study, we investigated the effects of adenovirusmediated XAF1 expression on liver tumor growth and tumor angiogenesis. We found that XAF1 could induce apoptosis and inhibit VEGF expression, tumor angiogenesis and tumor growth. Therefore, the restoration of XAF1 expression may be a new approach for liver cancer treatment.

## RESULTS

### The XAF1 is weakly expressed in HCC tissues and HCC cell lines

**Figure 1:**
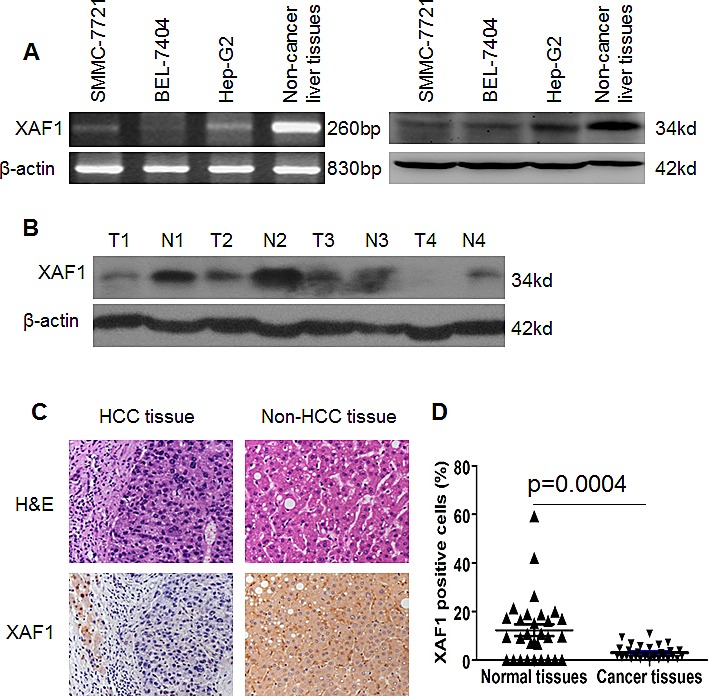
Expression of XAF1 in HCC cells and HCC tissues (A) The mRNA and protein expression of XAF1 in three human HCC cell lines and human non-cancer liver tissue detected by RT-PCR and Western Blot, respectively. (B) The expression of XAF1 was determined in human liver cancer tissues by Western Blot. T: liver cancer tissue; N: paired non-cancer liver tissue. β-actin was internal control. (C) XAF1 was expressed in the paired non-HCC tissues but not in HCC tissues. The HCC tissues and paired non-HCC tissues were suffered from H&E staining (*upper lane*)and XAF1 immunostaining (*bottom lane*) (magnification × 400). Representative images were shown. (D) Low expression of XAF1 in HCC tissues. The presented data are XAF1 positive expressing cells from 30 human HCC cases and non-cancer liver tissues. p < 0.004.

The mRNA and protein expressions of XAF1 were determined in 3 HCC cancer cell lines SMMC-7721, Bel-7404 and Hep G2, as well as 30 primary HCC cancer and paired non-HCC tissues. The result showed that the mRNA and protein expressions of XAF1 were lower in three HCC cell lines SMMC-7721, BEL-7404 and Hep G2 cancer cells compared non-cancer tissue of liver (Fig. 1A).

We further determined the expression of XAF1 in 30 human liver cancer tissues and paired adjacent non-cancer liver tissues. Western blot showed that XAF1 expression was lower in liver cancer tissues than that in the paired non-HCC tissues (Fig. 1B). IHC showed that XAF1 was expressed in non-HCC tissues but not in cancer tissues (Fig. 1C). XAF1 was localized in both cytoplasm and nucleus (Fig. 1C). Quantitative analysis of XAF1 expression showed that 66.7% (20/30) of non-HCC tissues strongly expressed XAF1, whereas only 16.7% (5/30) of liver tissues expressed XAF1 (Fig. 1D) (X^2^=15.43, P< 0.01). The results suggest that HCC tissues weakly expressed XAF1.

### Restoration of XAF1 expression inhibits proliferation and induces apoptosis of HCC cells

**Figure 2:**
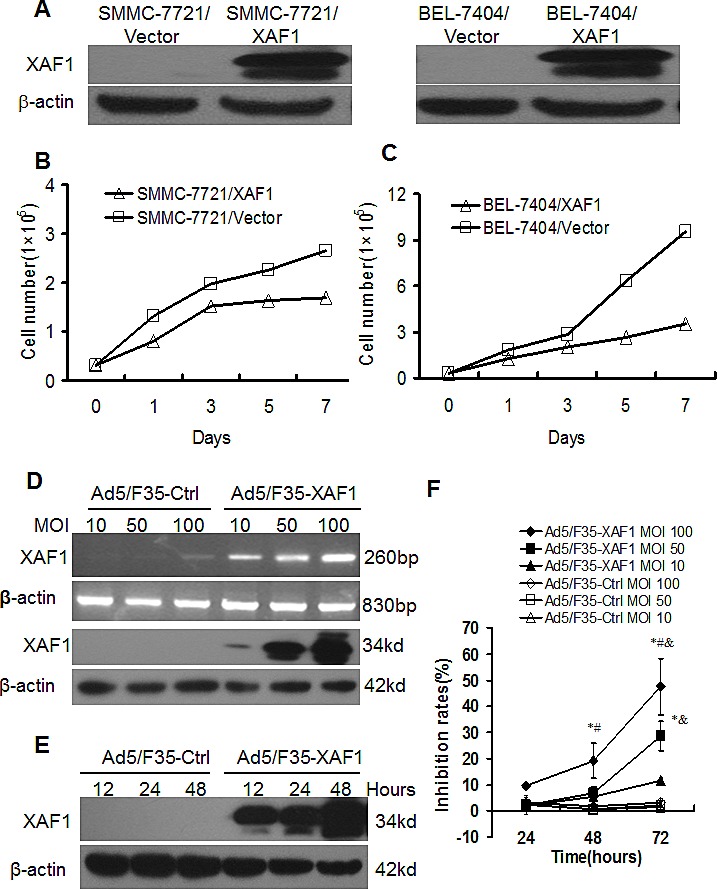
Overexpression of XAF1 inhibited cell proliferation of HCC cells (A) The overexpression of XAF1 protein in stable SMMC-7721/XAF1 transfectants and BEL-7404/XAF1 transfectants compared to the stable control transfectants detected by Western Blot. (B-C) Overexpression of XAF1 inhibited cell proliferation in stable SMMC-7721/XAF1 transfectants (B) and BEL-7404/XAF1 transfectants (C). The stable transfectants were cultured and counted in indicated time points. Data represent the means ± SD of three independent experiments.*p < 0.01, compared to control stable transfectants. (D-E) Ad5/F35-XAF1 virus infection increased XAF1 expression in HCC cells. SMMC-7721 cells were infected with Ad5/F35-XAF1 virus at indicated MOI for 48 hours. The mRNA expression of XAF1 was detected by RT-PCR. (F) Ad5/F35-XAF1 virus infection inhibited cell proliferation. SMMC-7721 cells were infected with Ad5/F35-XAF1 and control virus Ad5/F35-Ctrl at indicated MOI for 24, 48 and 72 hours. Cell proliferation was determined by MTT assays. The data are means ± SD of three independent experiments.

We determined the effect of restoration of XAF1 expression on proliferation of HCC cells *in vitro*. We established XAF1 stable transfectants in SMMC-7721 and BEL-7404 cells. Western blot confirmed that the XAF1 stable transfectants overexpressed XAF1 compared to the control transfectants (Fig. 2A). Quantitative analysis showed that the number of cells in the wells of SMMC-7721/XAF1 and BEl-7404/XAF1 stable tranfectants cultured were significantly lower than those in the wells of SMMC-7721/Vector and BEl-7404/Vector stable tranfectants cultured (Fig. 2B-2C). The results suggest that constitutive overexpression of XAF1 inhibits cell proliferation of liver cancer cells.

To determine the effects of transient expression of XAF1, we infected SMMC-7721 cells with Ad5/F35-XAF1 virus. We found that the mRNA and protein expressions of XAF1 were increased in a dose-dependent manner (Fig. 2D) and a time-dependent manner (Fig. 2E). MTT assay showed that infection of Ad5/F35-XAF1 virus resulted in inhibition of cell proliferation in dose- and time-dependent manners in SMMC-7721 cells, compared to Ad5/F35-Ctrl (Fig. 2F). These results show that the transient expression of XAF1 also inhibits cell proliferation of liver cancer cells.

### Ad5/F35-XAF1 virus induces apoptosis of HCC cells

**Figure 3:**
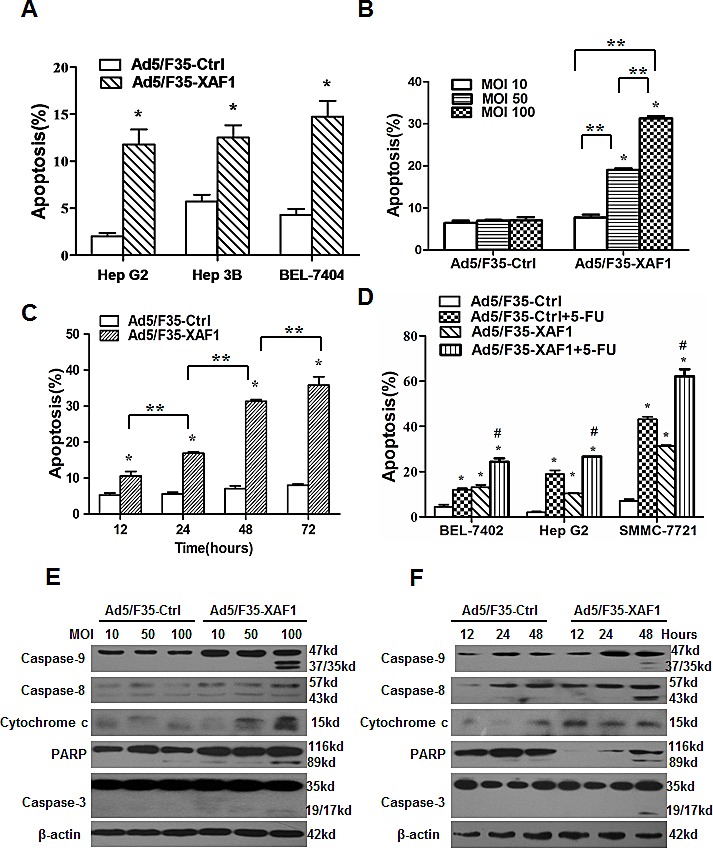
Restoration of XAF1 expression induced apoptosis in HCC cell lines (A) HCC cell lines were infected with Ad5/F35-XAF1 and Ad5/F35-Ctrl at MOI 100 for 48 hours. (B) SMMC-7721 cells were infected with Ad5/F35-XAF1 and Ad5/F35-Ctrl at indicated MOI for 48 hours. (C) SMMC-7721 cells were infected with Ad5/F35-XAF1 and Ad5/F35-Ctrl at MOI for indicated times. Apoptosis were determined by FACS. Data are means ± SD of three independent experiments. *p < 0.01, compared to the Ad5/F35-Ctrl treatment. (D) BEL7402, Hep G2 and SMMC-7721 cells were infected with Ad5/F35-XAF1 and Ad5/F35-Ctrl (MOI 50) alone or combined with 5-FU (25 μg/ml) for 48 hours. Apoptosis was determined by FACS. *p < 0.05 compared with Ad5/F35-Ctrl; #p < 0.05 compared with Ad5/F35-XAF1 alone group. (E-F) Infection of Ad5/F35-XAF1 activates intrinsic and extrinsic apoptotic pathways. SMMC-7721 cells were infected with Ad5/F35-XAF1 at indicated MOI for 48 hours (E) or at MOI 50 for indicated times. The expression of apoptosis-related proteins was detected by Western blot analysis. Reprehensive images were shown.

Next, we analyzed apoptosis induced by the infection with Ad5/F35-XAF1. The result showed that Ad5/F35-XAF1 virus induced apoptosis in four HCC cell lines (Fig. 3A). Furthermore, Ad5/F35-XAF1 virus induced apoptosis dose- and time-dependently in SMMC-7721 cells (Fig. 3B-3C). Next, we determined the effect of Ad5/F35-XAF1 on 5-FU-induced apoptosis. The results showed that the apoptotic rate were significantly higher in all 3 HCC cell lines treated with the combination of Ad5/F35-XAF1 and 5-FU than those in the cell treated with Ad5/F35-XAF1 and 5-FU alone (Fig. 3D), suggesting that Ad5/F35-XAF1 cooperates with 5-FU to induce apoptosis of HCC cells.

To determine the mechanisms of XAF1-induced apoptosis, we detected the expressions of apoptosis-related proteins 48 hours post-infection of Ad5/F35-XAF1 in SMMC-7721 cells by Western blot. Ad5/F35-XAF1 infection consistently induced the cleavage of caspase-9, 8, 3 and PARP and the release of mitochondrial cytochrome c into the cytosol in dose- and time-dependent manners (Fig. 3E-3F). However, Ad5/F35-Ctrl had no such effect (Fig. 3E-3F). The results indicate that Ad5/F35-XAF1 treatment induces apoptosis of HCC cells through activating the intrinsic and extrinsic apoptotic pathways.

### Ad5/F35-XAF1 inhibits tumor growth *in vivo*

**Figure 4:**
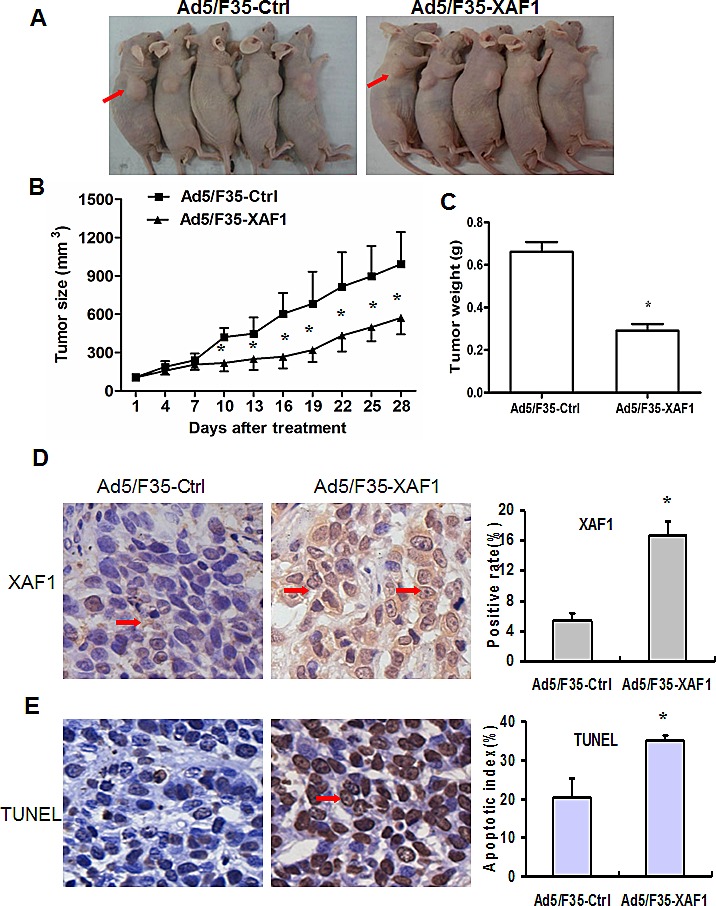
Ad5/F35-XAF1 inhibits tumor growth *in vivo* (A) SMMC-7721 cells were subcutaneously injected into the right back of female nude mice. When growing to approximately 100 mm^3^ −150 mm^3^, tumor received the injection of Ad5/F35-XAF1 and Ad5/F35-Ctrl virus at 1×10^9^ PFU/ at 3 sites for 5 days. Photos were taken from representative mice 4 weeks after treatment. (B) Tumor volume presented is means ± SD of five mice from (A). (C) Tumor weight was measured 4 weeks after treatment. The data presented are means ± SD of five tumors each group. *p < 0.05, compared to the Ad5/F35-Ctrl group. (D-E) Ad5/F35-XAF1 treatment increased XAF1expression and induces apoptosis *in vivo*. Tissue sections were suffered to XAF1 immunostaining (D) and TUNEL assay (E). (Original magnification × 400). Quantification of XAF1 expression and apoptotic index were described in “Materials and Methods.” Data presented are means ± SD of five mice. *p < 0.05, compared to the Ad5/F35-Ctrl group.

We further investigated the inhibitory effect of Ad5/F35-XAF1 on established subcutaneous tumors. Uninfected 1×10^7^ SMMC-7721 cells were injected *s.c.* into the right flanks of athymic nude mice to establish tumor. Single intra-tumor administration of Ad5/F35-XAF1 inhibited SMMC-7721 xenograft growth by approximately 48.7% at 4 weeks post-treatment, compared to Ad5/F35-Ctrl treatment. The mean tumor volume treated with Ad5/F35-XAF1 was smaller than that treated with Ad5/F35-Ctrl after 10 days of treatment (218.33 ± 66.62 mm^3^ *vs* 420.04 ± 72.25 mm^3^, P < 0.01), and the tumor volume was still significantly smaller than the groups until 4 weeks after treatment (570.73 ± 129.04 mm^3^ *vs* 992.44 ± 251.15 mm^3^, P < 0.01) (Fig. 4A-4B). The tumor weight in the Ad5/F35-XAF1 treatment group was smaller than that in Ad5/F35-Ctrl treatment group (P < 0.05). The average weight of xenograft tumor treated with Ad5/F35-Ctrl and Ad5/F35-XAF1 was 0.662 g ± 0.103 g and 0.291 g ± 0.070 g, respectively (Fig. 4C). The results demonstrate that Ad5/F35-XAF1 treatment inhibits tumor growth in HCC cells.

We then investigated the mechanisms by which Ad5/F35-XAF1 inhibited tumor growth. IHC showed that XAF1 expression was located in both cytoplasm and nuclei, and its expression was markedly increased in tumor tissues treated with Ad5/F35-XAF1 compared to that with Ad5/F35-Ctrl (Fig. 4D, *left panel*). Qualification analysis showed that the ratio of XAF1 positive staining was significantly higher in Ad5/F35-XAF1 treatment group than that in Ad5/F35-Ctrl treatment group (16.67% ± 1.85% *vs* 5.43% ± 0.96%, P < 0.01) (Fig. 4D, *right panel*). Accordingly, the apoptotic index in the tumors treated with Ad5/F35-XAF1 was significantly higher than that in the tumors treated with Ad5/F35-Ctrl group (35.22%±1.0 % *vs* 20.23% ± 5.14%, P < 0.01) (Fig. 4E). These results indicate that intra-tumor treatment with Ad5/F35-XAF1 significantly restores XAF1 expression and induces cell apoptosis *in vivo* and inhibit HCC xenograft tumor growth.

### Ad5/F35-XAF1 inhibits tumor angiogenesis by downregulating VEGF expression

**Figure 5:**
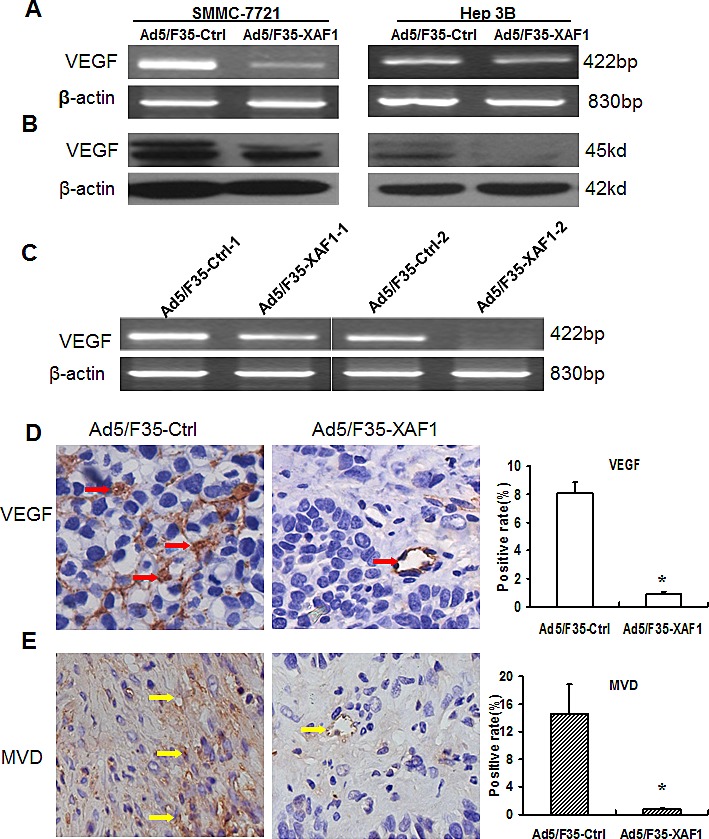
Ad5/F35-XAF1 inhibits VEGF expression and tumor angiogenesis (A-B) Ad5/F35-XAF1 inhibited VEGF expression in HCC cells. SMMC-7721 cells and Hep 3B cells were treated with Ad5/F35-XAF1 for 48 hours. The mRNA (A) and protein (B) expression of VEGF was determined by RT-PCR and Western Blot, respectively. (C) Ad5/F35-XAF1 inhibited mRNA expression of VEGF in xenografted tumors determined by RT-PCR. (D) Ad5/F35-XAF1 inhibited protein expression of VEGF in xenografted tumors determined by IHC. The data presented are ± SD of five mice. *p < 0.05, compared to the Ad5/F35-Ctrl group. (E). Ad5/F35-XAF1 tumor angiogenesis *in vivo*. Tumor angiogenesis was assessed by IHC with CD31 antibody on sections of tumors from mice treated with Ad5/F35-XAF1 or Ad5/F35-Ctrl. (original magnification × 400). Quantification of angiogenesis was described in “Materials and Methods.” The MVD was the average of the vessel counts obtained in the five sections of each group. Data presented are means ± SD of five mice. *p < 0.05, compared to the Ad5/F35-Ctrl group.

Previous results suggest that XAF1 decreased migration and tube formation of mouse endothelial cells [33]. VEGF plays a critical role in endothelial cells. We determined the effect of XAF1 on VEGF expression and found that Ad5/F35-XAF1 virus treatment markedly decreased mRNA and protein expression of VEGF in SMMC7721 and Hep3B cells (Fig. 5A-5B). RT-PCR result showed that mRNA expression of VEGF was significantly decreased in the tumor tissues treated with Ad5/F35-XAF1 compared to that in tumor tissues treated with Ad5/F35-Ctrl (Fig. 5C). IHC showed that protein expression of VEGF was much lower in the tumor treated with Ad5/F35-XAF1 than that in the tumor treated with Ad5/F35-Ctrl (Fig. 5D, *left panel*). The ratio of VEGF positive staining was significantly lower in the tumor treated with Ad5/F35-XAF1 than that in the tumor treated with Ad5/F35-Ctrl (0.91% ± 0.17% *vs* 8.12 % ± 0.74%, P < 0.01, Fig. 5D, *right panel*).

We further investigated the effect of XAF1 tumor *angiogenesis in vivo*, and determined the expression of PECAM-1/CD31, a well-established endothelial cell marker [39] in tumor tissues treated with Ad5/F35-XAF1 and Ad5/F35-Ctrl (Fig. 5E). IHC showed that positive staining of CD31 was markedly lower in the tumors treated with Ad5/F35-XAF1 than that in the tumor treated with Ad5/F35-Ctrl (Fig. 5E, *right panel* lane). Tumor-associated neovascularization as indicated by MVD was quantified. MVD was markedly lower in the tumors treated with Ad5/F35-XAF1 than that in the tumors treated with Ad5/F35-Ctrl (0.86% ± 0.05% *vs* 14.65% ± 4.24%, Fig. 5E, *left panel*). These results indicate that Ad5/F35-XAF1 inhibits VEGF expression and tumor angiogenesis *in vivo*.

### Ad5/F35-XAF1 treatment prolongs survival time of tumor-bearing mice

**Figure 6:**
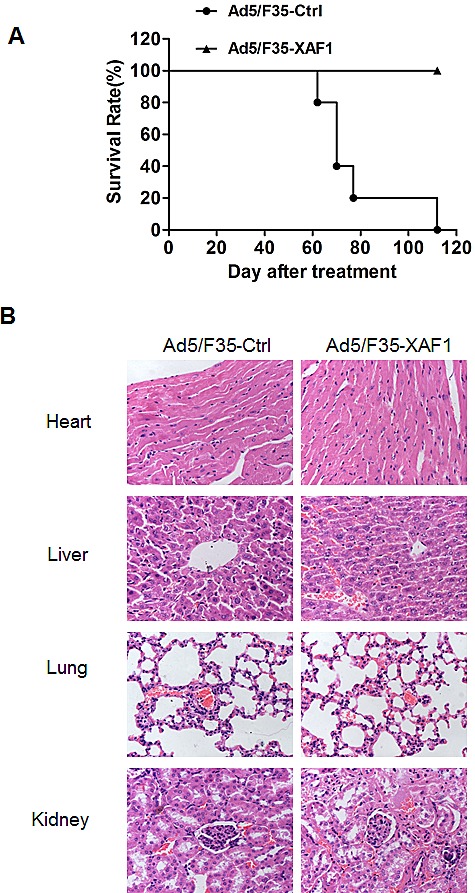
Ad5/F35-XAF1 prolonged the survival of mice (A) SMMC-7721 Tumor-bearing athymic nude mice were treated with Ad5/F35-XAF1 and Ad5/F35-Ctrl at 1×10^9^ PFU/ at 3 sites for 5 days. Mice were observed until the time point of natural death of all mice treated with Ad5/F35-Ctrl. (B) Histology analysis of main organs (heart, liver, lung and kidney) of mice treated with Ad5/F35-XAF1 and Ad5/F35-Ctrl 4 weeks after treatment (Original magnification × 200) are shown.

We finally assessed the effect of Ad5/F35-XAF1 on the long-term survival of tumor-bearing mice. The mice bearing tumor were treated with Ad5/F35-XAF1 and Ad5/F35-Ctrl, respectively. The survival time was observed for 4 months. The experimental end point was defined as the time when an entire group of mice died. Death was defined as natural death if tumor burden or tumor size was over 2 cm^3^. All mice died of natural death in Ad5/F35-Ctrl group, and no mouse died in Ad5/F35-XAF1 group by the end time point (110 days). Ad5/F35-XAF1-treated mice survived significantly longer than Ad5/F35-Ctrl-treated mice (P < 0.01). Our results suggest that Ad5/F35-XAF1 can prolong the survival time of tumor-bearing mice (Fig. 6A).

We also evaluated the safety of Ad5/F35 virus treatment *in vivo* by detecting the pathologic alterations of these four important organs from the mice 4 weeks after the treatment with Ad5/F35 virus. Histology analysis showed that the tissues of heart, liver, lung and kidney in all mice did not exhibit obvious pathologic changes 4 weeks after treatment (Fig. 6B). These results demonstrate the safety of Ad5/F35-XAF1 gene therapy.

## DISCUSSION

XAF1 is a tumor suppressor gene identified by two-hybridization in yeast [16]. The restoration of XAF1 expression has been shown to induce cell apoptosis in gastric and colorectal cancer cell lines and strengthen the apoptotic effects of chemotherapeutic drugs and TNF Related Apoptosis Inducing Ligand (TRAIL) [29, 30]. Gene therapy for recombinant adenovirus vector mediated XAF1 significantly suppressed tumor growth in gastric and colon cancer *in vitro* and *in vivo* [29-31]. Qi et al [40] also reported that XAF1 had potent antitumor activity when it was delivered by conditionally replicated adenovirus vector ZD55. In this study, we have shown, for the first time, that the restoration of XAF1 expression inhibited tumor growth and suppressed tumor angiogenesis in HCC both *in vitro* and *in vivo*.

Our previous studies have shown that XAF1 is weakly expressed in human gastric, colon and pancreatic cancer tissues [20, 23, 30]. A recent result showed that weak expression of XAF1 was associated with androgen deprivation resistance in prostate cancer [41]. The weak expression of XAF1 has been shown to be associated with portal vein tumor thrombi (PVTT), preoperative AFP level, tumor size, and recurrence of liver cancer[42]. The low expression of XAF1 was linked to apoptosis resistance of liver cancer [28]. Similarly, in this study, we also found that mRNA and protein expression levels of XAF1 were very low or undetectable in three HCC cell lines and HCC tissues compared to those in the adjacent non-cancer tissues. These results suggest that weak expression of XAF1 may play a role in the development of HCC [43].

In this study, we further demonstrated that the restoration of XAF1 could inhibit cell proliferation and induce apoptosis *in vitro* and *in vivo* in HCC cells. XAF1 is identified as XIAP binding protein and inhibits the function of XIAP anti-apoptosis. We found that Ad5/F35-XAF1 treatment induced the cleavage of caspase-3,-8,-9 and PARP but also increased the release of cytochrome c. Caspases are major proteins which execute cell apoptosis. Caspase-8 and caspase-9 are initial factors in extrinsic and endogenous apoptotic pathways. Cytochrome c is an important factor involved in mitochondria apoptotic pathway (intrinsic pathway). Our results suggest that Ad5/F35-XAF1 induces apoptosis of liver cancer cells through both endogenous and exogenous pathways, supporting our previous reports that XAF1 induces apoptosis in gastric and colon cancer cells [29, 30].

In this study, one new finding is that XAF1 could inhibit tumor angiogenesis in HCC. HCC has been shown to be a highly vascular tumor, and increased vasculature can lead to tumor rupture [44]. Angiogenesis (of new microvessel formation) is essential for the growth and progression of HCC because it enables the delivery of oxygen and nutrients [45]. Angiogenesis is regulated by angiogenic factors, such as VEGF and angiopoietins, which can be secreted by some tumor cells [46]. VEGF has been demonstrated as a central regulator of the angiogenic process in physiological and pathological conditions[47]. VEGF, also known as vascular permeability factor, stimulates the proliferation of endothelial cells through specific tyrosine kinase receptors [47]. VEGF is an important factor to evaluate the angiogenetic degree of tumor tissue. Previous results have shown that VEGF is strongly expressed and localized predominantly to cancer cells in HCC tissues, and its expression was strongly correlated with MVD and tumor size [13]. Therefore, the inhibition of angiogenesis has become a novel therapeutic choice [11, 48, 49]. In this study, we found that Ad5/F35-XAF1 treatment significantly decreased the expression of VEGF in HCC cell lines and tumor tissues. Furthermore, Ad5/F35-XAF1 treatment significantly inhibited MVD in HCC xenograft tissues. This result suggests that XAF1 suppressed angiogenesis *in vivo*. A study reported that XAF1 delivered by ZD-55 vector suppressed proliferation of mouse endothelial cells *in vitro* [33]. Our results demonstrate that XAF1 inhibits HCC cancer growth via suppressing VEGF expression and angiogenesis.

In this study, we used a recombinant adenovirus Ad5/F35 vector to mediate XAF1 expression. Ad5/F35 is a chimeric adenoviral vector which the fiber type 5 is replaced by fiber type 35 in adenovirus type 5 [50, 51]. The infective efficiency of Ad5/F35 virus is higher than adenovirus type 5 in most human cell lines such as hematopoietic cells, stem cell, lymphocytes and cancer cells [52, 53]. Although a previous study showed that adenoviruses may has a problem of delivery [54], we found that the infective efficiency of Ad5/F35-XAF1 virus was high in HCC cells. Therefore, Ad5/F35-XAF1 may also be considered as useful tool to study the role of XIAP. Many drugs have been developed to inhibit XIAP. For example, co-treatment of PI-103 or 17-AAG and TRAIL decreased XIAP and enhanced apoptosis [55]. Isorhapontigenin downregulates XIAP [56]. CDKI-73 decreased XIAP and synergizes with fludarabine for cancer inhibition [57]. The specificity of these drugs may be further confirmed by experiments with Ad/F35-XAF1 in the future.

In summary, our study demonstrates that the restoration of XAF1 expression induces tumor cell apoptosis and inhibits tumor angiogenesis. The XAF1 may be a promising candidate for HCC gene therapy.

## MATERIALS AND METHODS

### Cell lines and tissue samples

Human HCC cell lines SMMC-7721, BEL-7404, BEL-7402 (Shanghai Institutes for Biological Sciences, Shanghai, China), HepG2, Hep3B and human embryonic kidney cells (HEK293T) (ATCC, Manassas, USA) were maintained in DMEM medium (Gibco, CA, USA) containing 10% fetal bovine serum(FBS), (Gibco, CA, USA), 100U/ml penicillin and 100μg/ml streptomycin. All cell lines were maintained at 37°C and 5%CO_2_. The 293T cell line was used for the construction and amplification of Ad5/F35 vectors. Paired tumor and normal liver tissues were obtained from 30 patients who underwent surgical treatment in Ruijin Hospital. All cell lines were tested for mycoplasma by a PCR method (Stratagene) and all cell lines were mycoplasma negative. Tissues were snap frozen in liquid nitrogen and stored in −80°C. The frozen sections were examined to ensure that tumor specimens contained more than 70% malignant cells and normal specimens were free of tumor before proceeding to RNA and protein extraction. The study was approved by the Ethics Committee of Ruijin Hospital.

### XAF1 plasmids and transfections

The XAF1 cDNA expression vector pcDNA3.1-XAF1 has been described previously [29]. A 300-bp fragment XAF1 Coding DNA Sequence (CDS) was subcloned into pcDNA3.1 in the antisense orientation. The XAF1-antisense plasmid was used as control stable transfection experiment. Cancer cells were transfected with pcDNA3.1-XAF1 and XAF1-antisense plasmids using lipofectamine 2000 (Invitrogen, Carlsbad, CA). Stable transfectants were selected in 1000μg/ml Geneticin (G418). Stable clones overexpressing XAF1 were confirmed by Western blot analysis. The stable clonies were maintained in RPMI 1640 medium containing 200 μg/mL G418 for further studies.

### Construction and infection of recombinant adenovirus

A 740bp fragment of the XAF1 CDS was cut with EcoR I and BamH I and then subcloned into adenovirus vector p-shuttle plasmid PDC316 to generate PDC316-XAF1. PDC316-XAF1 plasmid was co-transfected with framing plasmid pBHG-fiber5/F35 into 293T cells. Recombinant adenovirus Ad5/F35-XAF1 was generated by screening and purification according to the method described previously [31]. The control adenovirus Ad5/F35-Control (Ctrl) was also generated using similar methods as described above. HCC cell lines SMMC-7721, BEL-7404, BEL-7402, Hep G2 and Hep 3B were infected with these three types of recombinant virus at different multiplicity of infection (MOI) and at different time points, respectively.

### Cell proliferation assay

Cancer cells were seeded into 96-well plates at 1×10^4^ cells/per well. After 24 hours, cells were infected with Ad5/F35-XAF1, Ad5/F35-Ctrl at different MOIs (10, 50 and 100) and at different time intervals (24, 48 and 72 hours). Non-infected cells were the control group. MTT (1 mg/mL) (Sigma, St. Louis, Mo) was added to the cells and incubated for additional 4 hours at 37°C. The supernatant fluid was removed, and 100 μL of dimethylsulfoxide (DMSO) (Sigma) was added to each well. The absorbance (OD value) was measured using a micro enzyme-linked immunoabsorbent assay reader (Thermo Scientific, Waltham, MA) at the wavelength of 570 nanometers (nm). The results were presented as cell viability. The inhibition rate of cell proliferation was calculated according to the following equation: Inhibition rate of cells = (1-OD_Ad5/F35_/OD_control_) ×100%.

### Apoptosis assay

HCC cells were seeded in 12-well plates at a density of 1×10^5^ cells/well and infected with different MOIs of adenovirus for 48 hours or with different time points (for 12, 24, 48 and 72 hours). Hep G2, BEL-7402 and SMMC-7721 cells were infected with Ad5F/35 virus (MOI 100) alone or combined with 5- Fluorouracil (5-FU) at 25 μg/mL for 48 hours. Cell apoptosis was determined by Flow cytometry (FCM) using Annexin V-FITC/PI double staining (BD Bioscience, San Jose, CA). Apoptosis was also determined by a terminal deoxynucleotidyl transferase biotin-dUTP nick end labeling (TUNEL) (Roche, Mannheim, Germany) assay according to the manufacturer's instructions. The xenograft tumor tissue sections from nude mice were fixed into 10% formalin. After deparaffinized and rehydrated, the tissue slides were added into the TDT enzyme and label solution (1:9) for an hour. Then, the tumor sections were incubated with POD (anti-fluorescein antibody). Staining was visualized using diaminobenzidine. Cells with brown nuclei staining were defined as apoptotic cells by light microscopy. The percentage of apoptotic cells was assessed in 5 randomly selected fields viewed at 400 × magnification. The apoptotic index (AI) was calculated as number of apoptotic cells/total number of nucleated cells ×100%.

### Reverse-transcription polymerase chain reaction

Total RNA was extracted from human HCC and paired non-cancer tissues, HCC cell lines and xenograft tumors from nude mice using Trizol (Invitrogen, Carlsbad,CA), respectively. One microgram of total RNA was used for complementary DNA synthesis (Reverse Transcription System, Promega, Madison, WI). Polymerase chain reaction (PCR) was run with a cycle at 95°C for 3 minutes and then 30 cycles at 94°C for 45 seconds, 57°C for 45 seconds, 72°C for 45 seconds and a final extension at 72°C for 10 minutes using 2×Taq PCR Mastermix (Tiangen, Beijing, China). The sequences of specific primers were as following: *XAF1* Forward primer 5'-TCCGGAATTCATGCTCCACGAGTCCTACTG-3', reverse primer 5'-ACGCGTCGACAAACTC TGAGTCTGGACAAC-3', 260bp; *VEGF* Forward primer 5'-CTTGCTGCTCTACCTCCAC3', reverse primer 5' AAATGCTTCTC CGCTCTG-3', 422bp; *β-actin* Forward primer 5'-ATCTGGCACCACACCTTCTACAA TGAGCTGC-3', reverse primer 5'-CGTCATACTCCTGCTTGCTGATCCACATCTGC-3', 830bp.

### Western Blot

Cells were lysed in RIPA buffer and PMSF. Protein samples (20μg) were electrophoresed on 6%~12% denaturing sodium dodecylsulfate (SDS) gels and transferred to PVDF membrane (Bio-Rad, Hercules, CA). The blots were incubated with specific primary antibodies as follows: Goat anti-XAF1 polyclonal antibody was purchased from Santa Cruz (Santa Cruz, CA). XIAP monoclonal antibody (mAb) was purchased from Stress Gene Biotechnologies Corp (Victoria, BC, Canada). Caspase-3,-8,-9 mAb antibodies and poly–(ADP-ribose)-polymerase (PARP) polyclonal antibodies were from Cell Signaling Technology Corp (Beverly, MA). Cytochrome c and β-actin were from Sigma (Saint Louis, MO). Primary antibody signals were detected using either horseradish peroxidase-conjugated second antibodies (Santa Cruz,CA). Anti-β-actin antibody was an internal loading control. Antigen-antibody complexes were visualized by the ECL system (Amersham Biosciences, Piscataway, NJ).

### Adenovirus Mediated XAF1 Gene Therapy in Xenograft Mice Model of HCC

Five- to six-weeks old female BALB/c nude mice (Special Pathogen Free, SPF) were bred in the Animal Experimental Centre of Shanghai Institutes for Biological Sciences (Shanghai, China). SMMC-7721 cells (1×10^7^ SMMC-7721) were injected subcutaneously (*s.c.*) into the right flanks of mice. When the size of tumor reached 100 −150 mm^3^ approximately, mice were randomly assigned to experimental group in which Ad5/F35-XAF1 were injected into tumor masses at 3 sites with 1×10^9^ plaque forming units (PFU) and to control group that received injection of Ad5/F35-Ctrl into tumors. Each group had 5 mice. The virus was injected into tumor every other day with a total of seven times. The experiment was repeated 3 times. Tumor size was measured using a caliper every three days after injection until experimental end time points. Tumor volume (V) was calculated according to the following formula: V (mm^3^) =1/2 ab^2^ (a: relatively shorter diameter, b: relatively longer diameter). Animals were euthanized 28 days after treatment, and their tumors were weighed and harvested for histology analysis, immunohistochemistry (IHC) and Western blot analysis. To evaluate the safety of Ad5/F35 virus treatment, four important organs, heart, liver, lung and kidney, were harvested from the mice injected with Ad5/F35 virus. To investigate the survival time of the xenografted mice treated with Ad5/F35-XAF1, other two groups of xenografted mice (5 mice/group) were also treated with Ad5/F35-Ctrl and Ad5/F35-XAF1 respectively as the same methods above. The end point was until an entire group of mice died.

### Immunohistochemistry

Tumor tissues were fixed in 10% formalin. Tumors were embedded in paraffin and stained with hematoxylin and eosin (H&E). The tissue sections were deparaffinized, rehydrated and boiled in 0.01M sodium citrate for antigen retrieval. The endogenous peroxidase activity was quenched. The sections were incubated with anti-XAF1 (1:500, abcam), anti-VEGF (1:250, abcam) and anti-CD31 (1:50, abcam, Cambridge, MA) overnight at 4°C. Tumor sections were incubated with biotinylated secondary antibodies, streptavidin_biotin complex (Dako, Glostrup, Denmark). Staining was visualized using diaminobenzidine. Representative photos were taken with a Nikon Eclipse E800 microscope equipped with a Nikon DXM1200 digital camera (Nikon instruments, Melville, NY, USA).

XAF1 and VEGF staining were recorded as the ratio of positively stained cells to all tumor cells in five different areas at × 400 magnification. MVD was evaluated according to the method described previously [39]. MVD was the average of the vessel counts (CD31 positive staining) obtained in the three sections. Areas of the highest neovascularization were chosen, and microvessel counting was performed at ×200 magnification in three chosen fields. Any immunoreactive endothelial cell or endothelial cell cluster that had been distinctly separated from adjacent microvessels was considered a single countable vessel. The results regarding angiogenesis in each tumor were expressed as the absolute number of vessels/0.74 mm^2^ (× 200 field). In all assays, matched isotype control antibodies were used and found to be unreactive in all cases.

### Statistical analysis

Data are presented as the means ± SD. The significance of the difference between groups was evaluated with the Student's t-test or one way variant analysis (ANOVA) by using SPSS15.0 software. *P* < 0.05 was considered significant. The chi-square test was used to analyze the difference in expression of XAF1 in human HCC samples. The Kaplan–Meier method was used to analyze survival time of tumor-bearing mice.
